# CMV Colitis in Immunocompetent Patients: 2 Cases of a Diagnostic Challenge

**DOI:** 10.1155/2016/4035637

**Published:** 2016-04-12

**Authors:** Maria Paparoupa, Viola Schmidt, Helgard Weckauf, Huy Ho, Frank Schuppert

**Affiliations:** ^1^Department of Gastroenterology, Endocrinology, Diabetology and General Medicine, Klinikum Kassel, Mönchebergstraße 41-43, 34125 Kassel, Germany; ^2^Institute of Pathology, Klinikum Kassel, Mönchebergstraße 41-43, 34125 Kassel, Germany

## Abstract

CMV infections are generally thought to be opportunistic by immunosuppression. Many literature cases though indicate that CMV infections can be also observed in immunocompetent patients. We present an unusual case of an extensive concentric benign stenosis due to CMV colitis and a case of coexistence with Crohn's Disease, both observed in nonimmunosuppressed individuals. The right diagnosis was set after implementation of multiple unsuccessful treatment strategies. Our purpose is therefore to familiarize clinicians involved with the diagnosis and treatment of gastroenterological diseases with this entity.

## 1. Introduction

CMV infections are generally thought to be opportunistic in patients with immunosuppressive diseases like HIV/AIDS, underlying malignancies, and organ- or bone marrow-transplantation and patients under treatment with chemotherapeutics or steroids [1–4]. However a rapidly rising number of literature cases worldwide indicate that CMV infections can be also observed in immunocompetent individuals, causing primarily gastrointestinal manifestations, like ulcerative colitis, pseudopolyps, or tumors, and ischemic and hemorrhagic enterocolitis [5–9]. The first case reporting verification of CMV colitis in an immunocompetent patient was published in 1992 [10]. Since then, particular underlying conditions are linked with a high risk of CMV colitis in apparently nonimmunocompromised patients, like chronic renal insufficiency and hemodialysis, aging, coinfection with bacterial gastrointestinal infections, and food allergy [11–19]. A notable connection between CMV colitis and colitis ulcerosa stands out in the literature [20–25], while only a single reported case correlates the first one with Crohn's Disease [26]. We present an unusual case of a persistent benign colon stenosis due to CMV colitis and a case of coexistence with Crohn's Disease, both observed in nonimmunosuppressed individuals. The right diagnosis was set after rigorous diagnostic procedures and implementation of multiple unsuccessful treatment strategies.

## 2. Cases Description

**Figure 1:**
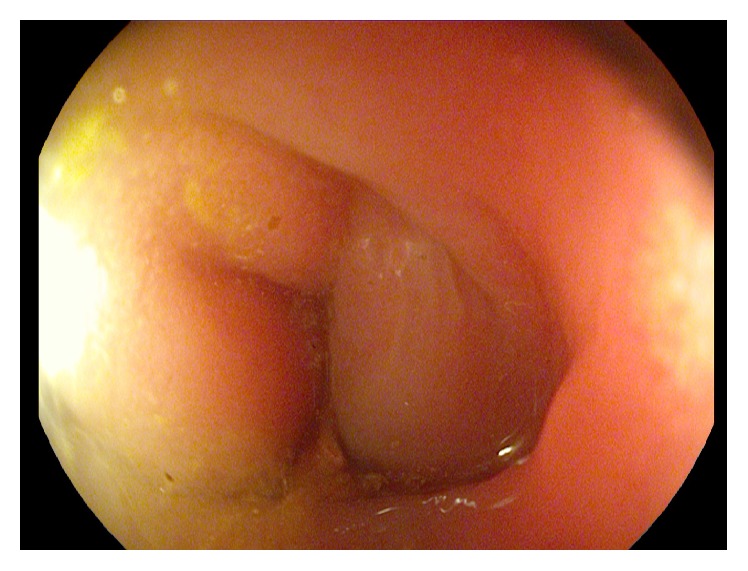
Persisting stenosis 40 cm from anus almost occluding colon lumen. The colonoscope could not be advanced beyond the mass.

**Figure 2:**
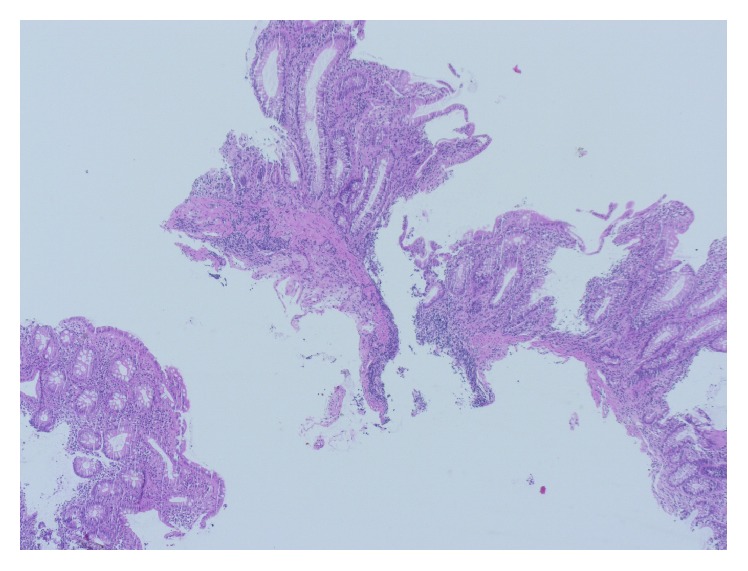
Light microscopy presenting biopsies of colonic mucosa with chronic inflammation and disorders of mucosal architecture (H & E, 100x).

**Figure 3:**
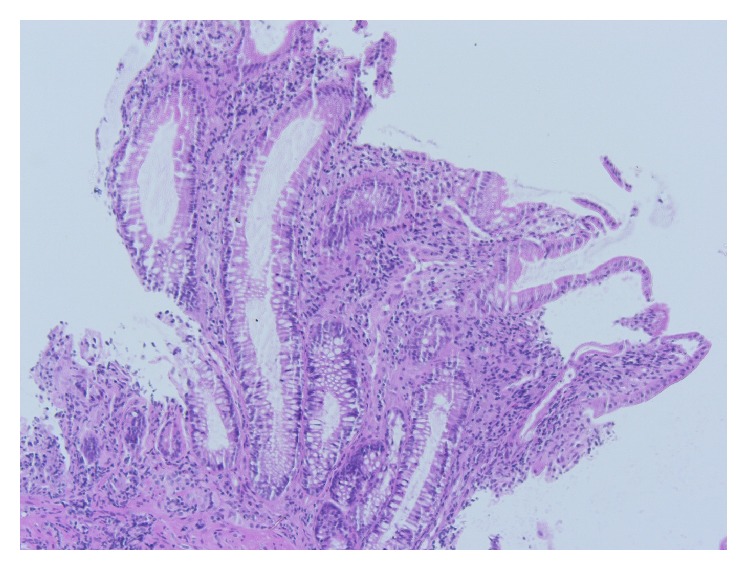
Histologic picture 2 in higher analysis (H & E, 200x).

**Figure 4:**
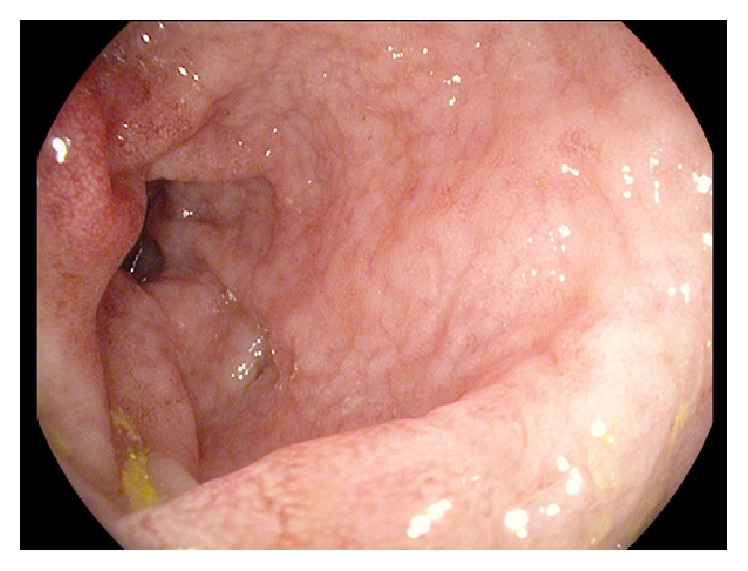
Colon stenosis slightly detectable in control colposcopy one month later.

A 77-year-old man presented to our Department of Gastroenterology with subileus symptoms and blood spots on his stool. COPD and laparoscopic cholecystectomy several years ago were reported as his medical history. A partial colonoscopy revealed multiple sigma diverticula with signs of inflammation and the patient was treated with oral mesalazine (Salofalk Granu-Stix 2000 mg/day) and antibiotics (ceftriaxone and metronidazole) for 2 weeks, leading to discrete relief of his symptoms. Two months later a control colonoscopy was performed; edema and erythema of mucosa were not present any more, but a persisting stenosis 40 cm from anus was almost occluding colon lumen (Figure 1). The colonoscope could not be advanced beyond the mass. All sigma diverticula presented unobtrusively and new biopsies were taken from the stenosis site. Histologic analysis confirmed a chronic inflammatory mucosal alteration and lymphofollicular hyperplasia of submucosa compatible with diverticula-associated colitis (Figures 2 and 3). By unclear distinctive stenosis of colon lumen and histological signs of chronic inflammation, atypical manifestation of IBD (inflammatory bowel disease) was hypothesized and immunosuppressive therapy with steroids was initiated, without clinical response. As last step of our diagnostic strategy, we performed serological and microbiological tests, in order to exclude gastrointestinal pathogens. Surprisingly, CMV-IgM Titer was positive and CMV-IgG Titer found greater than 200 RE/mL. After receiving this result, antiviral treatment with ganciclovir (Cymevene 600 mg/day) was initiated. A month later colon stenosis was slightly detectable and patient's subileus symptoms were no more present (Figure 4).

**Figure 5:**
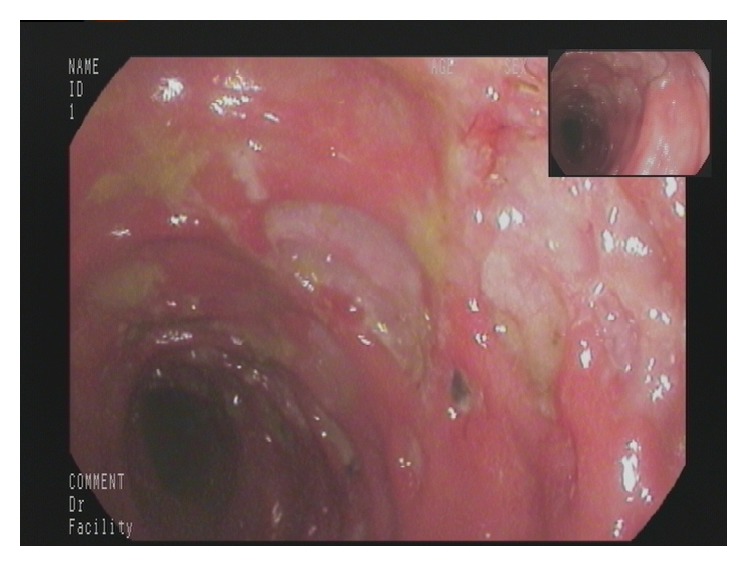
Multiple ulcerative lesions and hemorrhagic colitis.

**Figure 6:**
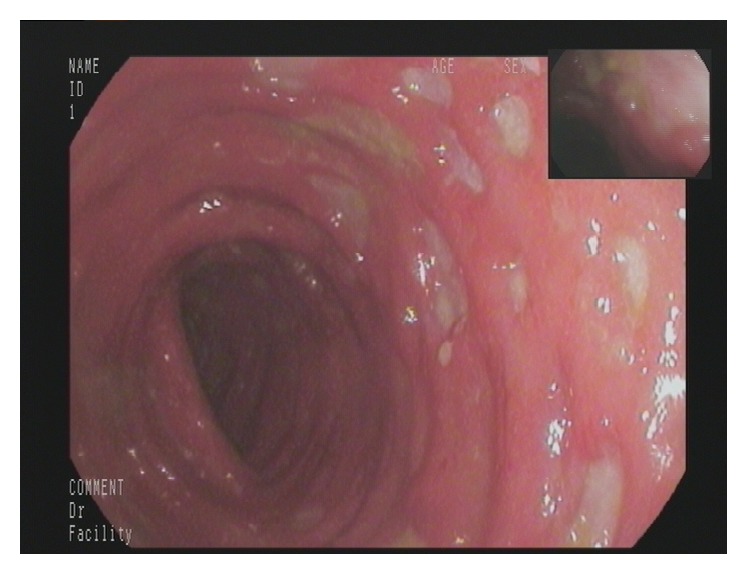
Endoscopic findings remained unchanged despite immunosuppressive therapy.

**Figure 7:**
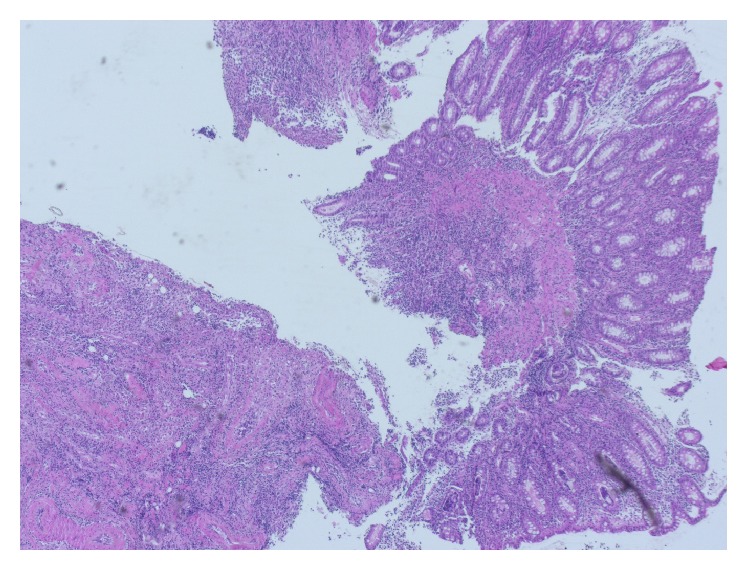
Highly active inflammation of colonic mucosa with an ulcerative lesion on the bottom of the picture (H & E, 100x).

**Figure 8:**
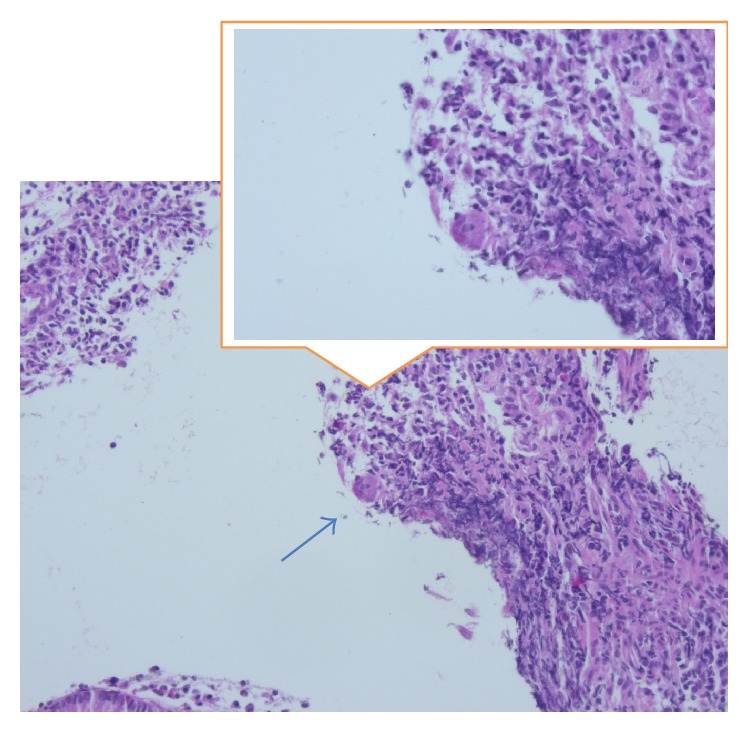
Virus infected cell at the edge of an ulcerous defect, known as “owl eye” cell, marked with an arrow and detailedly shown in the window (H & E, 400x).

In the second case, a 66-year-old man with Crohn's Disease in remission complained about diffuse abdominal pain, diarrhea, and weight loss starting 2 weeks ago. A high colonoscopy revealed multiple ulcerative lesions and hemorrhagic colitis leading to the diagnosis of acute Crohn's Disease exacerbation (Figure 5). We initiated immunosuppression with corticosteroids, with no clinical response, and then escalated to azathioprine. In the control colonoscopy, several days under immunosuppressive treatment, endoscopic findings remained unchanged (Figure 6). A new course of multiple step colon biopsies was conducted; multiple deeply reaching ulcers and lymphoid aggregates in the submucosa were detected (Figure 7). After a rigorous microscopic examination of histological samples, a typical intracellular inclusion of CMV virus known as “owl eye” was surprisingly detected on the border line of an ulcerative lesion (Figure 8). Elevation of CMV-IgM and IgG Titer in serum confirmed the diagnosis of CMV colitis and our patient was treated with intravenous ganciclovir (10 mg/kg/day), leading to complete clinical remission.

## 3. Discussion

These are two unusual cases of CMV colitis, manifested in immunocompetent patients, who presented to our Department for Internal Medicine and Gastroenterology over the last year. In both cases, patient symptoms and endoscopic findings imitated other more common conditions, like sigma diverticulitis and acute exacerbation of Crohn's Disease. This was the main reason for the delayed diagnosis. The implementation of multiple unsuccessful therapeutic strategies forced us to search further, without actually having CMV colitis in mind. In both cases the right diagnosis was set incidentally after positive CMV serology or typical histologic finding of “owl eye” cell. Our purpose is therefore to awaken clinicians involved with the treatment of gastroenterological diseases on the entity of CMV colitis in nonimmunocompromised patients.

In the first case, CMV colon infection presented as a persisting stenosis, which was initially almost occluding colon lumen. A similar case of an extensive colonic stenosis due to CMV colitis has been already described from Novakova et al. [27] in a 2-month-old immunocompetent male, infected via breast milk. In this case, stenosis was persisting after antiviral therapy and corticosteroids were initiated. Our patient showed no clinical response under steroids, but healed after antiviral therapy. According to literature reports, colonic occlusion due to CMV infection can be so severe and persistent that surgical removal is the ultimate therapeutic option [28]. However, inflammatory hyperplastic tissue forms more frequently pseudopolyps or tumor masses and not an extensive concentric benign stenosis like in our case and is mostly observed in immunocompromised compared to in immunocompetent patients [5, 6, 29–31].

The second case presented with multiple ulcerative lesions and hemorrhagic mucosa in a patient with known Crohn's Disease in remission. Clinical manifestations and endoscopic findings were compatible with acute Crohn's Disease exacerbation, but immunosuppressive treatment was unsuccessful. Shahani reported a case where diagnosis of Crohn's Disease followed the diagnosis of CMV colitis, as extraintestinal manifestations of IBD complicated the clinical course and antiviral therapy failed to improve the gastrointestinal symptoms of the patient [26]. According to scientific literature, CMV colitis can induce an acute exacerbation of IBD in remission [24] or complicate preexisting active and more often steroids refractory colitis flare [28]. In conclusion, CMV infection should be ruled out in patients with IBD exacerbation before starting immunosuppression and, on the contrary, IBD should be suspected in immunocompetent patients with CMV colitis.
